# Annexin A2 in Inflammation and Host Defense

**DOI:** 10.3390/cells9061499

**Published:** 2020-06-19

**Authors:** Valentina Dallacasagrande, Katherine A. Hajjar

**Affiliations:** Department of Pediatrics, Weill Cornell Medicine, 1300 York Avenue, New York, NY 10065, USA; vad2010@med.cornell.edu

**Keywords:** annexin A2, inflammation, infection, adherens junction, angiogenesis, macroautophagy

## Abstract

Annexin A2 (AnxA2) is a multifunctional calcium^2+^ (Ca^2+^) and phospholipid-binding protein that is expressed in a wide spectrum of cells, including those participating in the inflammatory response. In acute inflammation, the interaction of AnxA2 with actin and adherens junction VE-cadherins underlies its role in regulating vascular integrity. In addition, its contribution to endosomal membrane repair impacts several aspects of inflammatory regulation, including lysosome repair, which regulates inflammasome activation, and autophagosome biogenesis, which is essential for macroautophagy. On the other hand, AnxA2 may be co-opted to promote adhesion, entry, and propagation of bacteria or viruses into host cells. In the later stages of acute inflammation, AnxA2 contributes to the initiation of angiogenesis, which promotes tissue repair, but, when dysregulated, may also accompany chronic inflammation. AnxA2 is overexpressed in malignancies, such as breast cancer and glioblastoma, and likely contributes to cancer progression in the context of an inflammatory microenvironment. We conclude that annexin AnxA2 normally fulfills a spectrum of anti-inflammatory functions in the setting of both acute and chronic inflammation but may contribute to disease states in settings of disordered homeostasis.

## 1. Introduction

Inflammation is defined as the local host response to injury caused by infectious or noninfectious agents; it results in elimination or compartmentalization of the inciting agent, clearance of necrotic cells, and repair of damaged tissue [1,2]. Initially, host cells recognize danger-associated molecular patterns (DAMPs) or pathogen-associated molecular patterns (PAMPs) through germline-encoded pattern-recognition receptors (PRRs) expressed mainly in monocytes, macrophages, neutrophils, and dendritic cells [1]. The four known classes of PRR molecules include transmembrane proteins, such as Toll-like (TLRs) and C-type lectin receptors (CLRs), as well as cytoplasmic proteins, such as retinoic acid-inducible gene (RIG)-like receptors (RLRs) and nucleotide-binding oligomerization domain-like receptors (NLRs) [3]. These pathways induce the release of proinflammatory cytokines, which increase vascular permeability, thus facilitating the extravasation of immune cells into the damaged tissue, and chemokines, which recruit supplemental immune cells that phagocytose and kill pathogens [1]. Inflammation that resolves within days is classified as acute, whereas chronic inflammation may persist for months to years. Chronic inflammation underlies the pathogenesis of an array of disease entities, including diabetes, asthma, arthritis, cancer, atherosclerosis, vasculitis, and inflammatory bowel disease.

The annexins are Ca^2+^-regulated, phospholipid- and membrane-binding proteins that are named after the Greek word “*annex*”, meaning to attach or bridge because of their ability to link membranes to other membranes or other structures [4]. All but one (A6) of the twelve annexin family members (A1–A11 and A13) identified in vertebrates have a highly homologous core domain (~30–35 kilodaltons) containing four multi-alpha helical repeats with potential Ca^2+^-binding activity and an N-terminal domain (~3 kilodaltons), which is specific for each family member [5,6]. The annexins are expressed ubiquitously throughout the phylogenetic tree and are evolutionarily ancient.

Annexin A2 (AnxA2) is one of the most extensively studied members of the annexin superfamily [6]. AnxA2 is produced by a wide spectrum of cell types, including endothelial, trophoblast, epithelial, and tumor cells, as well as innate immune cells, such as macrophages, monocytes, and dendritic cells. AnxA2 may exist in either monomeric or heterotetrameric form. The heterotetramer (A2•S100A10)_2_ is composed of two copies each of AnxA2 and protein S100A10, also called p11. AnxA2 is present in the cytoplasm and on cell surfaces, and its functions are largely location specific. On the endothelial cell surface, the (A2•S100A10)_2_ complex binds components of the fibrinolytic system, plasminogen and tissue plasminogen activator (tPA), accelerating the activation of the serine protease plasmin [7,8]. As the primary fibrinolytic protease, plasmin enables fibrin breakdown and angiogenesis [9,10]. Intracellularly, AnxA2 seems to fulfill many functions, including organization of specialized membrane microdomains, facilitation of vesicle budding, and regulation of additional membrane dynamic events such as fusion, endocytosis, endosomal biogenesis, and membrane repair [4,5,11,12,13,14,15,16,17,18,19,20,21].

**Figure 1:**
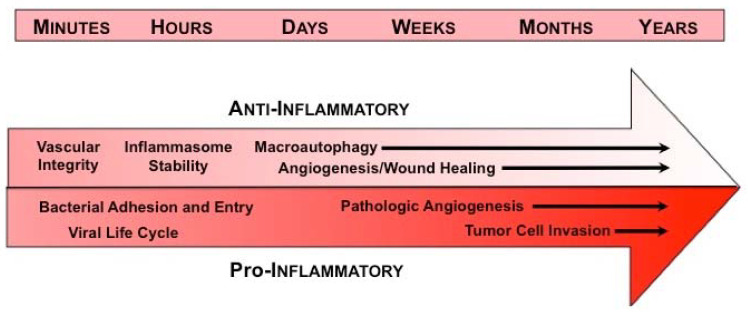
Annexin A2 in inflammatory responses—working model. Under homeostatic conditions (top arrow), AnxA2 plays an anti-inflammatory role in response to injury or infection. In the immediate response to injury, AnxA2 maintains vascular integrity, thereby preventing edema and extravasation of blood cells. Within minutes to hours, AnxA2 also protects internal membranes, such as those delimiting the lysosome, thus preventing inflammasome activation and tempering cytokine production. AnxA2 is also necessary for biogenesis of the phagophore, a double membrane structure that engulfs and destroys intracellular pathogens and allows the cell to adapt to environmental stresses in the process of macroautophagy. In the later stages of inflammation, AnxA2 likely promotes angiogenesis and wound healing by supporting cell surface fibrinolytic activity. In disease states, however (bottom arrow), AnxA2 may be co-opted to perform pro-inflammatory actions. On the plasma membrane, AnxA2 appears to serve as a site for bacterial adhesion and entry into cells, and may enable viral infection, replication, and release. In chronic inflammatory states, excessive angiogenesis that is sustained by AnxA2 may induce tissue damage, as in the diabetic retina, or may support the progression of cancer. In addition, some tumor cells may utilize cell surface AnxA2 to generate protease activity necessary for migration and metastasis within a pro-inflammatory micromilieu.

In view of this wide array of functions, we have developed a working model depicting the disparate anti- and pro-inflammatory roles of AnxA2 at the various stages of inflammation (Figure 1). In the initial phase, AnxA2 limits vascular permeability, thereby modulating recruitment of leukocytes and their subsequent release of inflammatory mediators. AnxA2 also supports internal membrane repair, thus modulating inflammasome activation, and participates in the biogenesis of the phagophore in autophagy, thereby facilitating the removal of pathogens and damaged organelles. Later, AnxA2 enables angiogenesis and tissue healing.

## 2. Annexin A2 and Infection

Infection results from the interaction of a pathogen (e.g., bacterium, fungus, or virus) with a host organism, leading to tissue invasion, proliferation of the pathogen, production of toxins, and cellular damage [22,23]. The first response of the mammalian host to infection is inflammation, mediated by the innate immune system, and is not specific to the inciting agent. The ensuing adaptive immune response is highly specific to the invading pathogen and typically provides long-lasting protection [23]. Annexin A2 fulfills a number of protective roles during pathogenic infection (Table 1).

In a model of *Klebsiella* pneumonia in mice, AnxA2 appeared to reduce infection-associated inflammation [24]. In *Anxa2^-/-^* mice infected intranasally with *Klebsiella* pneumonia, the level of pro-inflammatory cytokines was significantly elevated, and *Anxa2^-/-^* mice exhibited 100% mortality, versus 100% survival for *Anxa2^+/+^* mice at 50 h post injection. Additionally, peritoneal macrophages lacking AnxA2 showed worsening of the TLR4-triggered inflammatory response. These investigators concluded that AnxA2 has a role in limiting inflammation by promoting anti-inflammatory signals [24].

Annexin A2 also protects the host in a murine model of *Cryptococcal* infection. *Cryptococcus neoformans* is an encapsulated budding yeast that, unlike others, can replicate under acidic conditions; its virulence depends upon its interaction with host macrophages [25]. In this infection, *Anxa2^-/-^* bone-marrow-derived macrophages were less efficient than control macrophages at phagocytosing yeast cells, resulting in a lower frequency of non-lytic exocytosis. In addition, the *Cryptococcus* capsule was enlarged in *Anxa2^-/-^*-deficient macrophages, possibly impacting nonlytic exocytosis, and potentially reflecting the ability of AnxA2 to promote intracellular membrane interactions and vesicle adhesion to the cell membrane [6,26,27,28,29,30,31]. In vivo, mice lacking AnxA2 showed a lower survival rate when infected with *Cryptococcus*, reflecting a dysregulated inflammatory response [25].

On the other hand, there is evidence that cell surface annexin AnxA2 may be co-opted to facilitate infection by invading bacteria such as *Pseudomonas aeruginosa* [32], *Escherichia coli* [33], *Salmonella typhimurium* [34], and *Rickettsial* species [35]. *P. aeruginosa*, an organism that causes chronic pulmonary infection in patients with cystic fibrosis, anchors to AnxA2 on respiratory epithelial cells, undergoes internalization, and causes apoptotic cell death and release of pro-inflammatory cytokines [32]. In this instance, AnxA2 enables the initiation of productive infection.

Similarly, *E. coli*, particularly the EspL2 strain, reorganizes the cytoskeleton of the host epithelial cells with the participation of annexin A2, which is known to regulate actin dynamics at the bacterium–membrane contact site [36,37]. AnxA2 is recruited to the membrane adhesion site by *E. coli*, where its F-actin bundling activity is enhanced [33,38]. AnxA2 also promotes cytoskeletal rearrangements during host cell invasion by *Salmonella. S. typhimurium*, a common cause of gastroenteritis, uses the contact-dependent type 3 secretion system of the host cell to extensively remodel actin. AnxA2 is engaged in various mechanisms driven by actin rearrangement (e.g., endocytosis, cell–cell adhesion, and membrane ruffling) [34]. AnxA2 participates in reorganization of the actin cytoskeleton during *Salmonella* invasion; depletion of annexin A2 and S100A10 reduced bacteria invasion, probably because of the ability of these proteins to bind the large phosphoprotein AHNAK at the bacteria entry site [34].

In addition, annexin A2 acts as an adhesion receptor in *Rickettsial* infections [35]. In these disorders, bacteria adhere to and invade vascular endothelial cells in a manner that must be strong enough to overcome the shear stress of flowing blood. In human umbilical vein endothelial cells, immunofluorescence–confocal microscopy revealed colocalization of AnxA2 and *Rickettsial* organisms on the external face of the plasma membrane. The numbers of *Rickettsia* adherent to wild-type mouse brain microvascular endothelial cells was significantly increased compared to those associated with cells from AnxA2-null mice. In-vivo findings corroborated the hypothesis that host endothelial cell AnxA2 serves as an adhesion receptor for *Rickettsial* species. In fact, using a plaque assay and with confocal imaging, He et al. showed an increase in *Rickettsia* in circulating blood and a concomitant decrease on the endothelial cell surface in AnxA2-null mice [35].

Finally, annexin A2 appears to be co-opted during the life cycle of at least 13 human viruses. Reported roles include attachment, penetration by receptor-mediated endocytosis or direct membrane fusion, replication, assembly, and release [39]. For example, the (A2•S100A10)_2_ tetramer was identified as a central mediator in human papillomavirus (HPV) attachment and intracellular trafficking, leading to progression of genital cancers. Suppression of AnxA2 altered the entry of HPV into target cells, whereas antibodies against S100A10 did not have the same effect [40]. In addition, the infection was significantly reduced when the (A2•S100A10)_2_ complex was eliminated, a result that was not seen when S100A10 alone was deleted [41,42].

## 3. Annexin A2 and Regulation of Vascular Integrity

One of the earliest responses to inflammation is a loss of vascular integrity, which facilitates the invasion of innate immune cells into the affected tissue. The maintenance of blood vessel integrity is an active process that, if interrupted, can cause hemorrhage, edema, and progressive inflammation. Vascular permeability is regulated by endothelial–endothelial cell junctions [43]. Homotypic interactions between vascular endothelial cadherin (VE-cad), a major constituent of the adherens-type junction, are regulated by Src-mediated tyrosine phosphorylation of VE-cad, which induces opening of the junction [44,45,46].

Annexin A2 interacts directly with VE-cad and is essential to maintaining VE-cad within cell junctions [45]. AnxA2 and S100 A10 interact independently with VE-cad via actin filaments, and vascular endothelial growth factor (VEGF) treatment disconnects AnxA2 and actin from the VE-cad complex, increasing vasculature permeability. Phosphorylation of the adherens junctions can be induced by either loss of Src homology phosphatase 2 (SHP2) or destabilization of cholesterol drafts, both of which implicate annexin A2 in vascular integrity [45]. Annexin A2 also regulates tyrosine phosphorylation of VE-cad in the pulmonary microvasculature. Under hypoxia, *Anxa2^-/-^* mice, unlike control *Anxa2^+/+^* animals, showed a significant increase in Src-related phosphorylation of VE-cad. *Anxa2^-/-^*, but not *Anxa2^+/+^*, mice also displayed an acute inflammatory response with infiltration of neutrophils into the lung parenchyma and development of pulmonary edema. With VE-cad and SHP2, AnxA2 forms a complex, which is disrupted in the absence of AnxA2, thus preventing dephosphorylation of VE-Cad and inducing vascular leak [47].

In addition, in-vitro and in-vivo studies support a role for annexin A2 in maintaining the blood–brain barrier. Analysis of *Anxa2^-/-^* mice showed fewer tight junctions containing zonulin-1 and claudin-5 and fewer adherens junctions containing VE-cad. In cultured primary human brain microvascular endothelial cells, AnxA2 seems to promote F-actin and VE-cad interactions by binding Robo4 and contributing to its complex formation with paxillin. This process appears to be essential for maintaining vasculature integrity in the central nervous system [48].

## 4. Annexin A2 and Recruitment of Inflammatory Cells

It is likely that annexin A2 is important for the recruitment of some classes of leukocytes to sites of inflammation. AnxA2 may interact with CD44 in the chemotaxis of neutrophil-like cells in response to complement factor 5a in vitro, and anti-AnxA2 appears to block this activity in human neutrophils [49]. At the same time, the level of expression of AnxA2 in freshly isolated human neutrophils appears to be low compared to other circulating leukocytes, especially human monocytes and monocyte-derived macrophages, where it is highly expressed [50]. In fact, anti-AnxA2 IgG impairs cytokine-directed monocyte migration through the extracellular matrix [50] This migratory activity also requires tPA-dependent activation of plasminogen to the serine protease plasmin. In addition, an in-vitro wound healing assay revealed that loss of AnxA2 expression in intestinal epithelial cells led to increased cell-matrix adhesion via a β integrin, and reduced cell migration [51], but whether this mechanism applies to inflammatory cells is unknown. Nevertheless, the full extent of the actions of AnxA2 in inflammatory cell recruitment in vivo remains to be determined.

## 5. Annexin A2 in Inflammasome Dynamics

As an adaptive response, inflammation must be tightly regulated; inadequate inflammation can lead to persistent infection, whereas excessive or prolonged inflammation can cause longstanding disease, such as chronic arthritis, neurodegeneration, inflammatory bowel disease, or metabolic syndrome (atherosclerosis, type 2 diabetes and obesity) [52,53]. Inflammasomes are large intracellular protein complexes that sense inflammatory triggers in macrophages, monocytes, dendritic cells, neutrophils, and epithelial cells. They activate caspase-1 in response to both pathogens and host-derived signals of cellular stress including leakage of lysosomal cathepsins into the cytosol, damage to mitochondria, and production of reactive oxygen species [52].

As an adaptive response, inflammation must be tightly regulated; inadequate inflammation can lead to persistent infection, whereas excessive or prolonged inflammation can cause longstanding disease, such as chronic arthritis, neurodegeneration, inflammatory bowel disease, or metabolic syndrome (atherosclerosis, type 2 diabetes and obesity) [52,53]. Inflammasomes are large intracellular protein complexes that sense inflammatory triggers in macrophages, monocytes, dendritic cells, neutrophils, and epithelial cells. They activate caspase-1 in response to both pathogens and host-derived signals of cellular stress including leakage of lysosomal cathepsins into the cytosol, damage to mitochondria, and production of reactive oxygen species [52].

In humans with joint replacement devices, artificial articular surfaces may generate non-biodegradable wear debris particles, which, upon phagocytosis by dendritic cells, damage the endolysosomal limiting membrane. Damage to membranes leads to discharge of lysosomal cathepsins and H^+^ ions into the cytosol and activation of the NLRP3 inflammasome [54]. In the cytosol of dendritic cells exposed to wear debris particles, AnxA2 was profoundly downregulated, and both AnxA2 and S100A10 translocated to the endosome, to promote resealing of damaged membranes. These findings confirmed the hypothesis that AnxA2 is involved in endolysosomal membrane repair, thus limiting inflammasome activation [54].

On the other hand, upon infection with *Anaplasma phagocytophilum*, a *Rickettsial* organism, annexin AnxA2 null macrophages showed evidence of impaired inflammasome activation. Interleukin-1β secretion, caspase-1 activation, and NLRC4 oligomerization were all reduced compared to wild-type macrophages under the same conditions. Moreover, *Anxa2^-/-^*-infected mice were significantly more susceptible to infection than wild-type mice, indicating that AnxA2 helps this pathogen evade the NLRC4 inflammasome-based host defense system [55].

## 6. Annexin A2 and Macroautophagy

Once the innate immune cells have identified the pathogens or damaged cells during the inflammatory response, the process of macroautophagy may be initiated. Macroautophagy is a cell survival mechanism, in which certain cytoplasmic constituents (damaged organelles, misfolded proteins, or bacteria) are degraded in a lysosome-derived, double-membrane-enclosed vacuole, and then recycled to maintain cellular homeostasis [56,57,58]. In inflammatory macroautophagy, the process begins when a pre-autophagosomal membrane elongates and forms an autophagosome by engulfing a portion of the cytosol. Autophagosomes initially fuse with endosomes to form amphisomes, and then ultimately fuse with lysosomes to degrade their contents [59]. Macroautophagy is associated with a number of human pathologies including cancer [60], myopathies [61], diabetes [62], neurodegenerative processes [63], and infectious disease [63,64].

Annexin A2 participates in macroautophagy by interacting with the autophagy-related protein Atg16, especially in the biogenesis of Atg16L-positive vesicles. In primary dendritic cells, which have the highest rate of plasma membrane turnover and are crucial for immunosurveillance, proteomic analyses identified a population of vesicles positive for both AnxA2 and Atg16L. Comparison of cells from *Anxa2^+/+^* and *Anxa2^-/-^* mice demonstrated that AnxA2 facilitates Atg16L-positive vesicle fusion, a fundamental step in phagophore formation and elongation. Thus, AnxA2 appears to link Atg16L to membrane vesicles [15].

Macroautophagy is upregulated in response to a wide range of chronic inflammatory disorders and other stress conditions. These include persistent infections, such as tuberculosis, neurodegenerative disorders, such as Parkinson’s disease, and chronic inflammatory disorders, such as Crohn’s disease [65]. Under starvation conditions, for example, macroautophagy correlates with increased expression of annexin A2 in cultured cells and in mouse brain through a transcriptional pathway involving Jun N-terminal kinase (JNK) and c-Jun. Biogenesis of autophagosomes was enhanced in cells fed exogenous AnxA2 and abrogated in AnxA2-null mice. Autophagosome formation may be regulated by the effect of AnxA2 on actin-mediated trafficking of the transmembrane protein Atg9a, as AnxA2 and Atg9a were found to colocalize with actin filaments, leading to the conclusion that AnxA2 anchors actin on Atg9a-positive vesicles [66].

In another study, activation of autophagy during oxygen–glucose deprivation was reported to be influenced by AnxA2 in human retinal endothelial cells. Knockdown of AnxA2 attenuated the initiation of autophagy, reduced cell viability, and fostered cell apoptosis [67]. In another study, *Anxa2^-/-^* mice sustained a more severe *Pseudomonas aeruginosa* infection, which spread more readily to the lung and other organs. This appeared to correlate with an inability to clear the infection when annexin A2 is silenced and with the role of AnxA2 in regulating autophagosome formation through the Akt1–mTOR–ULK1/2 signaling pathway [68].

Macroautophagy is also central to the pathogenesis of osteoarthritis, a form of chronic joint inflammation. Autophagy may be protective in normal cartilage, as expression of ULK1, Beclin1 and LC3, the primary genes regulating the autophagy pathway, is decreased in osteoarthritic cartilage and chondrocytes [69]. In an in-vivo study with green fluorescent protein (GFP)-conjugated LC3 (GFP-LC3)-transgenic reporter mice, which allow one to monitor autophagy, there was a decrease in autophagic activity associated with a size and number reduction of autophagosomes [70]. These studies confirmed that age-related osteoarthritis correlates with autophagy, underlining a possible role for AnxA2 in this chronic inflammatory disorder.

## 7. Annexin A2 in Angiogenesis

Angiogenesis is the process by which endothelial cells, derived from pre-existing vasculature, proliferate and migrate to create new blood vessels. It is distinct from vasculogenesis, in which endothelial cells or their precursors coalesce to form new vasculature [71]. Under physiologic conditions, blood vessels undergo constant turnover, in which the rate of vessel degeneration is counterbalanced by the rate of blood vessel renewal. Insufficient angiogenesis is associated with myocardial infarction, stroke, preeclampsia, and neurodegeneration, whereas excessive vascular growth supports inflammation, obesity-associated disorders, development and spread of malignant tumors, and vascular-based ocular disorders such as diabetic retinopathy [72]. During inflammation, secreted pro-angiogenic factors promote neovascularization. The formation of new blood vessels, in turn, facilitates infiltration of innate immune cells, thus perpetuating the inflammatory process [73]. Additionally, upregulation of the inflammatory response caused by the persistence of the instigating agent may also facilitate angiogenesis through endothelial cell proliferation and migration [74].

Early in angiogenesis, pro-angiogenic signals activate endothelial cells, which migrate into the extracellular matrix, proliferate, elongate and create a lumen. The (A2•S100A10)_2_ complex, residing on the endothelial cell surface, converts plasminogen into plasmin, which can then activate a cascade of matrix metalloproteases. Matrix metalloproteases (MMP) can proteolyze components of the basement membrane, thus liberating endothelial cells and permitting their directed migration [72]. Based on these observations, it has been hypothesized that AnxA2 may be involved in excessive, pathological angiogenesis across a range of disease states [71].

Several in-vivo studies demonstrate a role for annexin A2 in postnatal angiogenesis. In the corneal pocket assay, for example, AnxA2-deficient mice displayed a decreased capability of generating new blood vessels in the cornea stimulated with fibroblast growth factor [75]. In wild-type mice, this response was blocked when mice were placed on a high-methionine diet; methionine is metabolized to homocysteine, which modifies AnxA2 by forming a covalent adduct with cysteine 8 in the N-terminal tail region. Interestingly, when hyperhomocysteinemic wild-type mice were treated with intravenous AnxA2, normal corneal angiogenesis ensued [76]. In the Matrigel implant assay, similarly, neovessel formation was significantly impaired in *Anxa2^-/-^* mice. Unlike *Anxa2^-/-^* mice, wild-type mice showed neovascularization of implanted Matrigel plugs. Addition of a peptide that mimics the N-terminal domain of annexin A2 and blocks tPA binding reduced the number of von Willebrand factor positive cells by 80%, whereas a scrambled control peptide had no effect [75].

In the oxygen-induced retinopathy (OIR) model of angiogenesis, 7-day-old neonatal mice are subjected to a 75% O_2_ environment for 5 days and then returned to room air (21% O_2_) for an additional 5-day recovery period. Immunohistologic analysis of retinas revealed a significant reduction of both neovascular tufts and tuft cell nuclei in *Anxa2^-/-^* mice. Under OIR conditions, the accumulation of fibrin in the perivasculature was extensive in *Anxa2^-/-^* retinas, but almost indiscernible in *Anxa2^+/+^* retinas. Treatment of pups with ancrod, a fibrinogen-depleting agent, upon return to room air almost completely eliminated fibrin in both groups of mice, while at the same time doubling the extent of neovascularization in *Anxa2^-/-^* but not *Anxa2^+/+^* mice. On the other hand, pups treated with tranexamic acid, an inhibitor of fibrinolysis, drastically increased fibrin deposition and lowered neovascularization in *Anxa2^+/+^* but not *Anxa2^-/-^* retinas. In addition, it was established that hypoxia inducible factor-1, a master hypoxia-responsive transcription factor, directly regulates AnxA2 gene expression and that AnxA2 binding with S100A10 promotes neovascularization by enhancing plasmin activation and fibrin remodeling. Together, these data confirmed that both fibrinolysis and angiogenesis were impaired under ablation of annexin A2 [77]. Similarly, AnxA2 appears to be upregulated by vascular endothelial growth factor, and blockade of AnxA2 in ischemic mice inhibits retinal neovascularization [78]. In choroidal neovascularization, moreover, the AnxA2-binding agent, TM601, a synthetic form of chlorotoxin, significantly suppressed neovascularization by inducing apoptosis in endothelial cells. [79]. Together, these studies indicate that the annexin A2 fibrinolytic system is a key regulator of angiogenesis.

## 8. Annexin A2 and Tumor Progression

The correlation between inflammation, angiogenesis, and cancer is well known. In fact, hypoxia-induced release of VEGF, a feature of both cancer progression and tumor angiogenesis, increases cellular proliferation, invasion and metastasis [74]. The involvement of annexin A2 in tumor progression may be related to the overproduction of plasmin on the surface of aggressive cancer cells, on the surface of endothelial cells, or both [80]. In addition, AnxA2 may facilitate the recruitment of pro-angiogenic inflammatory cells into the tumor microenvironment, which may in turn promote tumor progression.

Overexpression of annexin A2 has been observed in many malignancies, including aggressive breast cancer. AnxA2 was found to be elevated in stromal cells and epithelial cells of invasive ductal carcinoma (MDA-MB231) but not in a less aggressive cell line (MCF-7). MDA-MB231 cells, unlike MCF-7 cells, had the ability to activate plasminogen, leading to the hypothesis that plasmin generating capacity correlates with breast cancer cell migration and aggressiveness, implicating AnxA2 as a key mediator in metastasis. They also reported that blocking AnxA2 with angiostatin, a competitor for plasminogen binding on endothelial cell surface, or an anti-AnxA2 monoclonal antibody hindered MDA-MB231 invasion and migration. Immunohistochemical analyses of human breast cancer tissues revealed AnxA2 and elevated tPA on the surface of cancer cells, but not normal cells, as well as evidence of inflammation within the tumor. Quantitative analysis of the microvascular density showed a correlation between new blood vessel formation and the annexin A2 expression pattern [81,82,83]. Furthermore, immunoneutralization of AnxA2 impaired plasmin-mediated activation of MMP-2/9 in the breast tumor microenvironment [84]. Together, these data suggest that AnxA2 plays a pivotal role in breast cancer invasion and angiogenesis.

Annexin A2 expression is significantly elevated in glioblastoma, a highly vascularized and exceedingly aggressive brain tumor [85,86]. Intracerebral inoculation of canine glioma cells into the brains of athymic rats showed that AnxA2 is present in clusters of tumor cells surrounding dilated tumor vessels. Overexpression of AnxA2 correlated with high expression of VEGF and platelet-derived growth factor (PDGF), suggesting that the level of AnxA2 reflected invasiveness [87]. In addition, the involvement of AnxA2 in glioma angiogenesis was highlighted by the demonstration that tumors in wild-type mice displayed significantly higher microvascular density and more dilated blood vessels than tumors in *Anxa2^-/-^* mice [88].

Increased expression of AnxA2 appears to correlate with tumor invasiveness in renal cell carcinoma [89,90], hepatocellular carcinoma [91], colorectal cancer [92] and lung cancer [93]. These data suggest that AnxA2 may be a potential target in cancer therapeutics. In these latter studies, expression of AnxA2 has not yet been shown to correlate with activation of plasmin, as it has in breast cancer and glioblastoma. In colorectal cancer, upregulation of AnxA2 is regulated by TGF-β, which induces epithelial–mesenchymal transition [92]. In addition, silencing of AnxA2 in lung cancer stem cells in mice induced a reduction in tumor weight, which correlated with the loss of both β-catenin and S100A100, suggesting that AnxA2 may directly or indirectly regulate metastasis [93]. The mechanism for these effects, however, remains poorly understood.

## 9. Conclusions

Annexin A2 appears to have a spectrum of functions across a multitude of inflammatory disorders. In early infection, according to our working model, AnxA2 may support infection by acting as an anchor protein that promotes adhesion and internalization of bacteria and viruses, regulating actin dynamics at adhesion sites, and enabling virus assembly. AnxA2 may also help recruit leukocytes to some sites of inflammation. AnxA2 also maintains the integrity of adherens junctions early in the inflammatory process by regulating phosphorylation of VE-cadherin. Numerous studies demonstrate that AnxA2 is required for optimal endosomal membrane stabilization and autophagosome biogenesis and promotes membrane repair during inflammation and infection. Cell-surface AnxA2 in complex with S100A10, finally, is a key factor in both malignant and non-malignant angiogenesis, and it contributes to tumor cell invasion and metastasis within an inflammatory microenvironment. We conclude, therefore, that annexin A2 has a primarily anti-inflammatory role, although it occasionally facilitates pathogen activity. Many of these actions have been elucidated through the use of the *Anxa2^-/-^* mouse, which has become a useful tool for understanding the role of AnxA2 in vivo (Table 2). Taken together, these activities identify annexin A2 as a robust biomarker and potential therapeutic target.

## Figures and Tables

**Table 1:** Role of annexin A2 in pathogen–host cell interactions in infection.

Pathogen	AnxA2 role	References
**Bacteria**		
	**Anti-Inflammatory Actions**	
*Klebsiella pneumoniae*	Reduced cytokine response	[24]
	**Pro-Inflammatory Actions**	
*Pseudomonas aeruginosa*	Plasma membrane adhesion	[32]
*Escherichia coli*	Plasma membrane adhesion	[33,38]
*Salmonella typhimurium*	Host cell invasion	[34]
*Rickettsia australis*	Plasma membrane adhesion	[35]
**Fungus**		
	**Anti-Inflammatory Actions**	
*Cryptococcus neoformans*	Phagocytosis and exocytosis	[25]
	Pro-Inflammatory Actions	
none reported	---	---
**Virus**		
	**Anti-Inflammatory Actions**	
Human Papillomavirus	Attachment and intracellular trafficking	[40,41,42]
	**Pro-Inflammatory Actions**	
none reported	---	---

**Table 2:** Studies in *Anxa2-/-* mice revealing a role of annexin A2 in inflammation.

Action	Model System	Result in *Anxa2-/-*	References
Infection	*Klebsiella* pneumonia	Increased mortality	[24]
	*Cryptococcal* pneumonia	Increased mortality and enhanced inflammatory response	[25]
	*Rickettsia australis*	Increased bacteria in blood and reduced adhesion to vascular endothelial cells	[35]
Vascular integrity	Hypoxia	Increased vascular leak in lungs and skin	[47]
	Tracer injection in embryonic mice	Increased leakage of 10-kDA-dextran	[48]
Inflammasome Dynamics	*Anaplasmaphagocytophilum* peritonitis	Increased bacterial load, splenomegaly, and cytopenias	[55]
Macroautophagy	Starvation-induced autophagy	Abrogation of autophagosome biogenesis	[66]
	*Pseudomonasaeruginosa* pneumonia	Reduced survival, increased bacterial dissemination, and blunted autophagy	[68]
Angiogenesis	Cornea pocket assay	Reduced growth factor induced neoangiogenesis	[75]
	Matrigel implant assay	Reduced growth factor induced neoangiogenesis	[78]
	Oxygen-induced retinopathy	Reduced angiogenesis and fibrinolysis with fibrin accumulation	[77]
Tumor progression	Intracerebral glioma cell implantation	Increased tumor size and reduced vascularity	[88]
